# 

**Published:** 1919-01

**Authors:** 


### SUBJECT INDEX TO VOLUME LXXIII

### EVENT AND COMMENT

A New Type Dental Office, 402.

Annual Meeting National Dental Association, 501.

Artistic Prosthesis, 301.

Christmas in America, 551.

Christmas in Armenia, 551.

Death of Dr. William W. Belcher, 555.

Dental Educational Requirements in New York, 555.

Dental Hygienist Legislation. 203.

*Dental Review* Ceases Publication, 1.

Editorial Retirement of C. N. Johnson, 3.

Free Dental Clinic in Every Public School, 502.

Improvidence of Dentists, 251.

Is Real Preventive Dentistry a Practicable Possibility, 552.

Is the Present Practice of Dentistry Benefiting Humanity? 451.

Next Move in Dental Education, 101.

Plate Prosthesis, 5.

Sears' Impression and Bite Methods, 151.

Shall We Extirpate the Pulp, 53.

Something New in Dental Society Organizations, 351.

Standardizing Technical Dentistry, 201.

The Pulp Canal and Its Problems, 51.

Things to Do in 1920, 556.

Trouble at Vanderbilt, 401.

### FORMAL PAPERS

Advantages and Disadvantages of Reduced Silver in Dental Therapeutics. By U. G. Rickert, 534.

An Experiment that Failed. By C. N. Johnson, 521.

Amalgam Mixing, 133.

Army Medical Corps, 185.

Articulating Artificial Dentures. By O. A. Weiss, 267.

Artistic Prosthesis. By J. Leon Williams, 304.

Asepsis in Root Canal Operations. By E. S. Best, 174.

Care of the Eyes. By C. D. Wescott, 84.

Combination Impressions for Acurate Fitting Dentures. By Victor H. Sears, 153.

Complement Fixation Tests as Applied to Dental Practice. By A. Zabriski, 283.

Dentistry for the Masses, 478.

Dental Anesthesia. By Dr. A. F. Smith, 578.

Face Masks. By G. A. Comte, 388.

First-Line Trench in Dentistry. By Evangeline Jordan, 318.

Food Enzymes; Essential Factors of Complete Nutrition. By Thomas G. Read, 129.

Harrison Act, 131.

Howe's Root Canal Filling. By U. G. Rickert, 91.

Ideal Method of Brushing the Teeth. By W. J. Charter, 210.

Importance of Dental Services in the Hospitals. By A. A. Crocker, 316.

Impression Filler and Surface Separator. By Rupert E. Hall, 386.

Importance of the Septal Gingival Margins. By H. E. Friesell, 70.

Instruments and Technique to be used in Pulp Canal Operations. By E. S. Best, 113.

Itinerant Dental Instructors. By Julio Endelman, 274.

Malocclusion and Pyorrhea Alveolaris, 136.

Maxillo-Facial in the American Army. By V. P. Blair, M.D., 352.

New Root Filling. By W. Clyde Davis, 485.

On the "Flu"—A Theory. By S. N. Young, 126.

Opium must not be Prohibited, 438.

Oral Foci of Infection from the Dental Standpoint. By H. H. Schuhmann, 416.

Packing Vulcanite. By W. M. Randall, 94.

Partial Lower Compound Impressions with the Mouth Closed. By Samuel G. Supplee, 327.

Physiological Cost of Chewing, 435.

Physiological Factors as Related to the Peridental Membrane, Cementum and Bone in Tooth Movement. By Martin Dewey, D.D.S., 575.

Popular Education in Dietetic Economics. By A. L. Benedict, M.D., 335.

Preventive Dentistry. By Russell W. Bunting, 527.

Preventive Dentistry. By W. D. Tracy, 216.

Prevention of Diseases. By a Physician, 276.

Preventive Orthodontia. By R. J. Wenker, 462.

Preventive Orthodontia in Early Childhood and the Management of Children. By H. A. Pullen, 220.

Primi-Dentures. By F. W. Frahm, 74.

Relationship of Dentistry to Health Conservation. By Harvey J. Burkhart, 58.

Relation of Teeth, Tonsils and Intestinal Toxemias to Diseases of the Eye. By George H. Bell, M.D., 503.

Retention of Full Dentures. By Rupert E. Hall, 16.

Retention of Full Upper and Lower Dentures. By Russell W. Tench, 370.

Roentgenographic Studies of Tissues Involved in Chronic Mouth Infections. By A. D. Black, 5.

Root Canal Operations, 271.

Root Canal Filling and Health. By Edgar D. Coolidge, 164.

Rootless, Natural Teeth. By A. G. Gardner, 132.

Some Dental Facts, 431.

Some Problems in Histology, Physiology and Pathology to be Solved. By John S. Marshall, 204.

Specialization in Dentistry, 411.

Starting a Dental Practice, 482.

Technique of Administering Nitrous Oxide to the Average Adult. By J. P. Henahan, 360.

Technique for Constructing Artificial Dentures. By M. M. House, 32.

The Council of Pharmacy and Chemistry—Present and Future. By W. A. Puckner, 256.

The Higher and Better Education of the Dental Student. By Eugene S. Talbot, 454.

Toothbrush Exercise. By Sidney Rauh, 409.

Treatment and Filling of Root Canals. By F. W. Gethrow, 471.

Trench Mouth. By Guy A. Karr, 281.

Unwarranted Assumption of Professional Duties. By Julio Endelman, 123.

War Exhibit at the Recent Meeting of the A. M. A., 433.

What are the Chances that You Will Die Rich? By Richard D. Wyckoff, 253.

What is Dental Literature? By Garrett Newkirk, 377.

Why I Should Keep Accurate Records of Business Transactions, 586.

Young Patients and the Dentist, 95.

INDENTS

Aluminum Solder, 244.

A Man's a Man for a' That, 143.

American Creed, 441.

An Age of Quackery, 442.

Anesthetizing and Sterilizing the Gum Locally, 291.

Anesthetic for the Aged, 393.

An Iodine Lotion, 291.

Anticipant Dentures, 48.

Applying Iodine, 147.

A Profession or an Occupation, 44.

Articulator, A Natural, 98.

Are You Ready to Practice Modern Dentistry, 495.

Asepsis in Filling Root Canals, 488.

Barbed Broaches for Molars, 141.

Bleaching a Tooth, 145.

Canadian Foundation for Dental Research, 395.

Casting Inlays, 345.

Cement Destroyed by Alteration in Water Content, 195.

Charcoal and Ulcerative Stomatitis, 242.

Chewing Gum at the Front, 492.

Chinosol in Root Filling, 589.

Clasps for Partial Dentures, 47.

Conforming the Wax for Cast Dentures, 292.

Consolidation of Health Bodies, 140.

Country and City Dentist, 443.

Dental Granuloma Cause of Hiccough, 243.

Dental Hygienist, 489.

Dental Hygiene in Rochester, 491.

Dentistry and Cancer, 344.

Dental Reconstruction and Bolshevism, 547.

Dental War Souvenir, 146.

Desensitizing Surgical Margins, 589.

Devitalizing Pulps, 43.

Dichloramin-T Solution, 193.

Dietetic Value of Nuts, 187.

Drilling Cavities in Porcelain, 244.

Duplicating Plaster Casts, 291.

Education of a Dentist, 487.

Etching the Gold Inlay, 590.

Evils of Spit and Spitting, 139.

Extracting Caution, 590.

False Teeth and Cancer, 138.

Fatal Cocaine Poisoning, 142.

Flasking, 244.

Fracture of Lower Jaw, 139.

Full Denture Retention, 97.

Gateway of Sorrows, 343.

Handle for Small Investments, 592.

Harvard Surgical Unit, 347.

Infant Mortality in U. S., 392.

Influenza—A Precaution for Physicians, 99.

Iodoform in Root Fillings, 592.

Local Anesthesia for Children, 490.

Local Anesthesia, 440.

Malformed Teeth, 445.

Massachusetts Dental Hygiene Work, 492.

Metal Antagonizing Casts, 547.

Method of Denture Retention on Flat and Hard Surfaces, 294.

Method of Firmly Attaching Vulcanite to Aluminum, 391.

Mobilizing for Peace, 440.

Mouth Wash for Oral Suppurative Conditions, 488.

Need for More Consultations Among Dentists, 191.

Nervous Shock in Dentistry, 46.

Nickel-Copper Amalgam, 140.

Oral Hygiene, 546.

Oral Hygiene Inspection for N. Y., 491.

Our Next Duty, 142.

Passing a Strip Through Tight Contacts, 292.

Pathology and Prophylaxis of Pyorrhea, 591.

Perry Separation, 292.

Pepsondont, 292.

Polishing Ground Porcelain, 142.

Poor Impressions, 294.

Preparedness League, 100.

Preventing Plaster Expansion in Setting, 344.

Progressive or Retrogression, 144.

Pulp Canal Treatment, 445.

Pulp Protection, 141.

Red Cross Dentistry, 493.

Relation of Teeth to Ridge in Lower Dentures, 546.

Removing Plaster from Vulcanite Dentures, 346.

Repairing Pink Vulcanite, 142.

Replacing a Tooth on a Vulcanite Plate, 444.

Respiratory Diseases, 139.

Rockefeller Foundation, 97.

Root Resection, 293.

Science of Health, 393.

Sealing Arsenic in a Tooth, 141.

Securing Canal Broaches, 345.

Separating Tape, 140.

Serious but Ludicrous Accident, 494.

Setting Crowns and Bridges, 146.

Strassburg School Clinic, 490.

Strength of Epinephrin for Local Anesthesia, 394.

Support for Work in the Porcelain Furnace, 546.

Teething, 344.

That Precious American Soap, 589.

The Future of Dental Practice, 590.

The Point of View, 188.

Theory of Asthma, 345.

Therapy of Buccal Cancer, 496.

The Tooth Area, 42.

To Facilitate Grinding Tooth Substances, 590.

Tragedies of the Profession, 497.

Trench Mouth, 344.

Triumphs of Surgery, 190.

Use of Natural Teeth for Crowns, 44.

What Did I do to this Patient? 396.

What is Nickel-Silver? 391.

While We Sleep, 143.

BIOGRAPHIC

Beecher, Dr., 488.

Bell, G. H., M.D., 503.

Benedict, A. L., M.D., 335.

Best, Elmer S., 113, 174, 446.

Black, Arthur D., 5.

Blair, V. P., 352.

Bloom, Theo., 489.

Bunting, R. W., 527.

Burkhart, H. J., 58.

Charbonneau, Dr., 98.

Charter, W. J., 210.

Colyer, Stanley, M.D., L.D.S., 567.

Comte, G. A., 388.

Coolidge, E. D., 164.

Crocker, A. A., 316.

Crymes, F. J., 291.

Cunningham, George, 245.

Davis, W. Clyde, 485.

Dewey, Martin, D.D.S., 575.

Dowsley, J. F., 499.

Endleman, Julio, 123, 274.

Essig, N. S., 42, 48, 99.

Farr, R. E., 441, 491.

Frahm, F. W., 74, 141, 147, 142, 242, 292, 546, 547, 590.

Fones, A. C., 490.

French, A. B., 182.

Friesell, H. E., 70.

Fuqua, V. H., 589.

Gardner, A. G., 132.

Gethrow, F. W., 471.

Guilford, S. H., 200.

Haag, Flora N., 103.

Hall, Rupert E., 16, 386.

Heckert, Dr., 142.

Henehan, J. P., 360.

Hoolar, M. W., 243.

House, M. M., 32.

Hulin, M., 44.

Jenkins, N. S., 549.

Johnson, C. N., 3, 521.

Jordan, Evangeline, 318.

Karr, Guy A., 281.

Leak, Wm. H., 491.

Lever, S., 140.

McClintock, Gray, 445, 499.

McCoy, John, 390.

McManus, James, 390.

Marshall, J. S., 204.

Mayo, W. J., M.D., 344.

Mellanby, May, 231.

Murray, R., 547.

Morehead, F. B., 488.

Nelson, J. F., 292.

Newkirk, Garrett, 377.

Newling, R. L., 590.

O'Hara, F. S., M.D., 403.

Ottolengui, R. E., 444.

Prinz, H., 195, 244.

Puckner, W. A., 256.

Pullen, H. A., 220.

Randall, W. M., 94.

Rauh, Sidney, 409.

Read, Thomas G., 129.

Rickert, U. D., 91, 534.

Roy, Maurice, 591.

Royer, Dr., 100.

Rosenow, E. C., M.D., 557.

Rudin, N., 291.

Savage, W. H., 147.

Schuhmann, H. H., 416.

Sears, V. H., 153.

Seccomb, W., 394.

Shepardson, Dr., 45.

Simpson, E. G., 590.

Smedley, V. C., 44, 146.

Smith, Dr. A. F., 578.

Spaulding, W. H., 142.

Supplee, S. G., 327.

Talbot, E. S., 454.

Taliferro, Dr., 139.

Teetzel, Captain, 294.

Tench, R. W., 370, 546.

Thomas, E. H., 293.

Tracy, W. D., 216.

Vaughan, V. C., 139.

Voelker, Chas. C., 588.

Walters, W. S., 445.

Webster, A. E., 191, 292.

Wenker, R. J., 462.

Weiss, O. A., 267.

Wescott, C. D., 84.

Wheeless, R. L., 47.

Williams, J. Leon, 304.

Wilson, W. B., 142.

Wyckoff, R. D., 253.

Young, N. S., 126.

Zabriskie, A., 283.

### ANNOUNCEMENTS

American Institute of Dental Teachers, 196, 587.

American Red Cross, 500.

Assistants Wanted in Forsythe Infirmary, 247.

Association of Military Dental Surgeons, 397.

Build Now Movement, 295.

Callahan Memorial, 247.

Delta Sigma Delta, 392.

Dental Protective Association, 550.

Illinois State Society, 196.

Indiana State Society, 196.

International Society of Prosthodontists, 397.

Kentucky State Society, 148, 196.

McManus, Dr. James, 390.

Michigan State Society, 148.

Modern Dentistry for the Laity, 196.

National Association of Dental Faculties, 397, 500.

National Association of Examiners, 397.

National Dental Association, 397.

National Association Meeting at New Orleans, 447.

New Jersey State Examiners, 500.

Northern Ohio Dental Society, 196.

New York State Society, 196.

New York State Examinations, 500.

Officers Michigan Society, 295.

Ohio State Society, 550.

Program, National Association, 397, 447.

Preparedness League, 48, 148, 248, 297.

Psi Omega Fraternity, 397.

Red Cross Notes, 349.

Report of French and Belgian Relief Work, 348.

Section Stomatology Program, American Medical Association, 296.

Texas State Society, 148, 196.

United States a Going Concern, 197.

Victory Loan, 151.

We Must Keep Faith, 249.

West Virginia State Society, 148.

Xi Psi Fraternity, 397.

OBITUARY

George Cunningham, 245.

### ANNOUNCEMENTS

---

### PREPAREDNESS LEAGUE

*Future Work of the League.* Although organized as a war emergency measure, conditions have so changed that there is a definite call for the League in a broader and more embracing sense. In fact, those who rightly read the signs of the times are united in the belief that the fullest usefulness of the organization is yet to come.

The identity of the League will, no doubt, be absorbed by the broader conception and we will be big enough to take up service for our profession throughout the allied nations. Should not the dentists of the United States and Canada, who have in reality gained greatly by the war, give every possible assistance to our brothers who have suffered loss of practice, home, money and even worse? Is there not a call to you through professional sympathy and the brotherhood of man to come forward in this, their hour of darkness and need and stretch forth a helping hand? Can you compute the wonderful influence thereby exerted toward establishing bonds that will live for all time?

Let us see the vision and act without delay. Let us keep alive the great principle upon which the League was founded and which has made it so great a power for good. Our National Dental Association needs such an organization to forward public service work in its name and to do such service for the welfare of the people as cannot be accomplished by a society whose activities are purely scientific in character. We must remember that the old order of things has been completely changed by conditions beyond our control, therefore, let us quickly adjust ourselves accordingly and meet these new conditions as we have so successfully met everything thus far.

Before this message shall have reached you, it is the hope of the officers of the League that this matter shall have crystallized and definite plans have been formed. We wish to call your attention to the excellent editorial in the December *Cosmos* which should be read by every member of the League. The outline of our future possibilities so well stated there should receive careful consideration from the profession and any suggestions will be gladly received at League Headquarters.

The above is merely a suggestion of one of our future activities but there are others of local as well as of general significance to be considered which will make the League more useful than ever.

*Our Home Service Work.* Director-General Tracy has sent a letter to all State Directors to co-operate with the Home Service Section of the Red Cross in caring for the families of soldiers and sailors in need of dental treatment. All members of the League are requested to take up this splendid work and by communicating with your State Director you will be given necessary instructions. Let us make a record that we will be proud of.

*Send in Reports.* All our members are earnestly requested to send in all reports of free dental service to the New York Headquarters. Hundreds of thousands of such operations have not been reported.

How many have you failed to report?

*Lantern Slides.* Those holding one or more sets of the lantern slides sent out from Headquarters last winter, will kindly communicate with the President and state whether it is desired to retain them or return them.

### American Institute of Dental Teachers

The next annual meeting of the American Institute of Dental Teachers will be held at Hotel Piedmont, Atlanta, Georgia, January 28, 29 and 30, 1919.

Papers on the teaching of war dentistry and an exhibit of war appliances will be the main features, and along with these will be the usual papers on teaching methods.

All persons interested are cordially invited.—Abraham Hoffman, *Secretary,* 381 Linwood Avenue, Buffalo, N. Y.

### Alumni Society of the Dewey School of Orthodontia

The Alumni Society of the Dewey School of Orthodontia will hold their next annual meeting in St. Louis, March 6, 7 and 8, 1919. The usual high standard of the meetings of this society will be maintained. All interested in Orthodontia are welcome. Address communications to Dr. George F. Burke, 741-43 David Whitney Bldg., Detroit, Michigan.

